# Time definition of reintubation most relevant to patient outcomes in critically ill patients: a multicenter cohort study

**DOI:** 10.1186/s13054-023-04668-3

**Published:** 2023-09-30

**Authors:** Aiko Tanaka, Yoshimitsu Shimomura, Akinori Uchiyama, Natsuko Tokuhira, Tetsuhisa Kitamura, Hirofumi Iwata, Haruka Hashimoto, Suguru Ishigaki, Yusuke Enokidani, Tomonori Yamashita, Yukiko Koyama, Naoya Iguchi, Takeshi Yoshida, Yuji Fujino

**Affiliations:** 1 Department of Anesthesiology and Intensive Care Medicine, Osaka University Graduate School of Medicine, 2-15 Yamadaoka, Suita, Osaka 565-0871 Japan; 2 Department of Intensive Care, University of Fukui Hospital, Yoshida, Fukui Japan; 3 Division of Environmental Medicine and Population Sciences, Department of Social and Environmental Medicine, Osaka University Graduate School of Medicine, Suita, Osaka Japan; 4 Department of Hematology, Kobe City Hospital Organization Kobe City Medical Center General Hospital, Kobe, Hyogo Japan; 5 Department of Pediatrics, Osaka University Graduate School of Medicine, Suita, Osaka Japan

**Keywords:** Reintubation, Extubation failure, Mechanical ventilation, Mortality, Intensive care

## Abstract

**Background:**

Reintubation is a common complication in critically ill patients requiring mechanical ventilation. Although reintubation has been demonstrated to be associated with patient outcomes, its time definition varies widely among guidelines and in the literature. This study aimed to determine the association between reintubation and patient outcomes as well as the consequences of the time elapsed between extubation and reintubation on patient outcomes.

**Methods:**

This was a multicenter retrospective cohort study of critically ill patients conducted between April 2015 and March 2021. Adult patients who underwent mechanical ventilation and extubation in intensive care units (ICUs) were investigated utilizing the Japanese Intensive Care PAtient Database. The primary and secondary outcomes were in-hospital and ICU mortality. The association between reintubation and clinical outcomes was studied using Cox proportional hazards analysis. Among the patients who underwent reintubation, a Cox proportional hazard analysis was conducted to evaluate patient outcomes according to the number of days from extubation to reintubation.

**Results:**

Overall, 184,705 patients in 75 ICUs were screened, and 1849 patients underwent reintubation among 48,082 extubated patients. After adjustment for potential confounders, multivariable analysis revealed a significant association between reintubation and increased in-hospital and ICU mortality (adjusted hazard ratio [HR] 1.520, 95% confidence interval [CI] 1.359–1.700, and adjusted HR 1.325, 95% CI 1.076–1.633, respectively). Among the reintubated patients, 1037 (56.1%) were reintubated within 24 h after extubation, 418 (22.6%) at 24–48 h, 198 (10.7%) at 48–72 h, 111 (6.0%) at 72–96 h, and 85 (4.6%) at 96–120 h. Multivariable Cox proportional hazard analysis showed that in-hospital and ICU mortality was highest in patients reintubated at 72–96 h (adjusted HR 1.528, 95% CI 1.062–2.197, and adjusted HR 1.334, 95% CI 0.756–2.352, respectively; referenced to reintubation within 24 h).

**Conclusions:**

Reintubation was associated with a significant increase in in-hospital and ICU mortality. The highest mortality rates were observed in patients who were reintubated between 72 and 96 h after extubation. Further studies are warranted for the optimal observation of extubated patients in clinical practice and to strengthen the evidence for mechanical ventilation.

**Supplementary Information:**

The online version contains supplementary material available at 10.1186/s13054-023-04668-3.

## Background

Mechanical ventilation is essential for life support of critically ill patients, and an adequate strategy is related to patient outcomes. As soon as the underlying disease that ultimately requires mechanical ventilation is controlled and the patient can maintain spontaneous breathing and sufficient oxygenation and ventilation, extubating and promptly liberating the patient from mechanical ventilation are imperative [1]. Despite standard weaning procedures, including spontaneous breathing trials (SBTs), 3–20% of patients require reintubation [2–5]. Post-extubation respiratory failure causes respiratory fatigue involving the diaphragm, which requires additional mechanical ventilation for recovery [6, 7], resulting in prolonged intensive care unit (ICU) and hospital stays, greater costs, and increased in-hospital mortality [8, 9]. A delay in reintubation may induce progressive clinical deterioration [10]; therefore, reintubation should be performed appropriately.

Reintubation has a significant impact on outcomes in patients requiring mechanical ventilation; however, the time definition of reintubation varies considerably in the literature. Current international guidelines provide reviews and recommendations based primarily on reintubation within 48 h [11, 12]. Conversely, large randomized controlled trials of weaning from mechanical ventilation have used various definitions of reintubation at different time points, including 48–72 h [13–18]. This discrepancy in the definition of reintubation may have important implications for patient outcomes and the development of guidelines for critically ill patients [19, 20]. Hence, the timing of reintubation, which has the greatest impact on patient outcomes, needs to be investigated, and the period of observation required after extubation needs to be defined.

In this study, we aimed to examine in detail the impact of reintubation on patient outcomes, including the time course from extubation to reintubation, in critically ill patients in a multicenter setting. Our findings will strengthen the evidence regarding mechanical ventilation and provide a strong scientific basis for future studies.

## Methods

### Study design

We performed a multicenter retrospective cohort study of mechanically ventilated adult patients who underwent extubation in ICUs between April 2015 and March 2021. We investigated data from the Japanese Intensive Care PAtient Database (JIPAD), a nationwide registry of critically ill patients. The JIPAD includes a large dataset with regular data checks similar to those of the Australian and New Zealand Intensive Care Society (ANZICS) Adult Patient Databases, based on a partnership agreement between the Japanese Society of Intensive Care Medicine and the ANZICS Center for Outcome and Resource Evaluation [21]. Patients 18 years of age or older who underwent mechanical ventilation and extubation at their first ICU admission were included. The exclusion criteria included tracheostomy before ICU admission, death, ICU discharge, or tracheostomy during the first mechanical ventilation episode, and missing data.

This study was conducted according to the STrengthening the Reporting of OBservational studies in Epidemiology (STROBE statement) [22] and the relevant guidelines and regulations. This study was approved by the Ethics Review Board of Osaka University Hospital (approval number: 21539), with a waiver for written consent for participation.

### Data collection

Details of sociodemographic data, comorbidities, severity, biochemical data at ICU admission, ICU treatment, and patient outcomes were recorded for all patients admitted to ICUs registered in the JIPAD. We extracted the baseline characteristics for each patient, including age, sex, body mass index, comorbidities (chronic heart failure, chronic respiratory failure, chronic liver disease, malignancy, immunodeficiency, and maintenance dialysis: [yes/no]), emergency admission (yes/no), surgical type of admission (yes/no), systematic diagnosis for ICU admission (cardiac, respiratory, gastrointestinal, neurological, sepsis, trauma, metabolic, hematological, renal/genitourinary, gynecological, other), acute physiology and chronic health evaluation (APACHE) II and III scores as indicators of disease severity on ICU admission, and data within 24 h after ICU admission (incidence of acute kidney injury [yes/no], ratio of arterial oxygen partial pressure [PaO_2_] to fractional inspired oxygen [FiO_2_], and Glasgow Coma Scale [GCS] score < 8 [yes/no]). Chronic liver disease was defined as cirrhosis or liver failure. Acute leukemia/multiple myeloma, lymphoma, and metastatic cancer were considered malignancies. Immunosuppression and acquired immunodeficiency syndrome were classified as immunodeficiencies. We collected the following processes of care and patient outcomes: implementation of venovenous (VV) or venoarterial extracorporeal membrane oxygenation (ECMO) (yes/no), use of renal replacement therapy (RRT) (yes/no), noninvasive respiratory support including noninvasive ventilation and high-flow nasal cannula (yes/no), duration of first or total mechanical ventilation, reintubation (yes/no), tracheostomy during ICU stay (yes/no), length of ICU or hospital stay, and ICU or in-hospital mortality (yes/no). The use of high-flow nasal cannula was recorded from April 2018. Reintubation was defined as the re-implementation of invasive mechanical ventilation within 120 h after extubation. For patients who underwent reintubation, the time from extubation to reintubation and the number of reintubation episodes were recorded.

### Outcomes

The primary outcome of this study was in-hospital mortality. The secondary outcome was ICU mortality.

### Statistical analyses

Categorical variables were summarized as numbers (percentages), and the chi-square test or Fisher’s exact test was used for comparison. Continuous variables were described using the median and interquartile range (IQR), and the Mann–Whitney U-test or Kruskal–Wallis test were used for two-group or multigroup comparisons, respectively. For all extubated patients, the cumulative probability of reintubation was described using Gray’s method, with death as the competing risk. Univariable and multivariable Cox proportional hazard analyses were performed to describe the relationships between reintubation and ICU and in-hospital mortality. Age, sex, comorbidity chronic heart failure and chronic respiratory failure, APACHE III score, PaO_2_:FiO_2_ and GCS at admission, duration of first mechanical ventilation, and use of noninvasive respiratory support were added to the multivariable model of mortality to adjust for confounding factors regarding mortality and reintubation. Taking into account its simplicity for use as a clinical observation period or research outcome, patients who underwent reintubation were divided according to the number of days between extubation and reintubation during the first reintubation episode [23], forming five groups. For the patients who were reintubated, the hazard ratios (HRs) and 95% confidence intervals (CIs) were investigated on each date of reintubation after extubation using univariable and multivariable Cox proportional hazard analyses. A two-sided p-value less than 0.05 was considered statistically significant. All statistical analyses were conducted using R version 4.2.2 (2022, R Foundation for Statistical Computing, Vienna, Austria).

## Results

### Extubation in critically ill patients

**Fig. 1:**
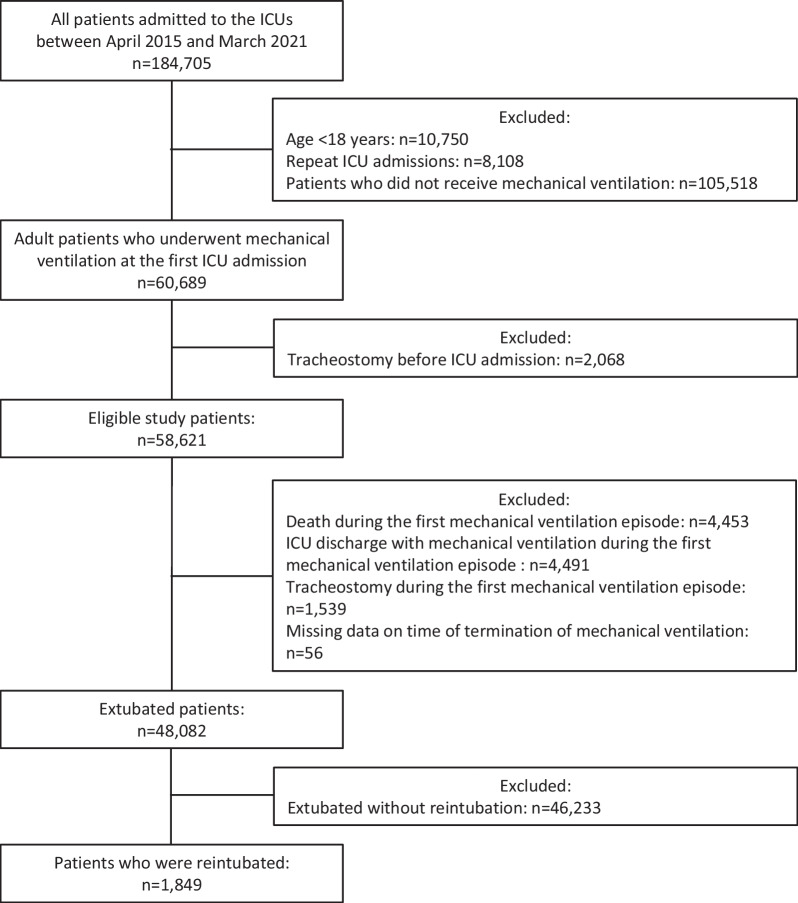
Patient inclusion flowchart. ICU, intensive care unit

**Fig. 2:**
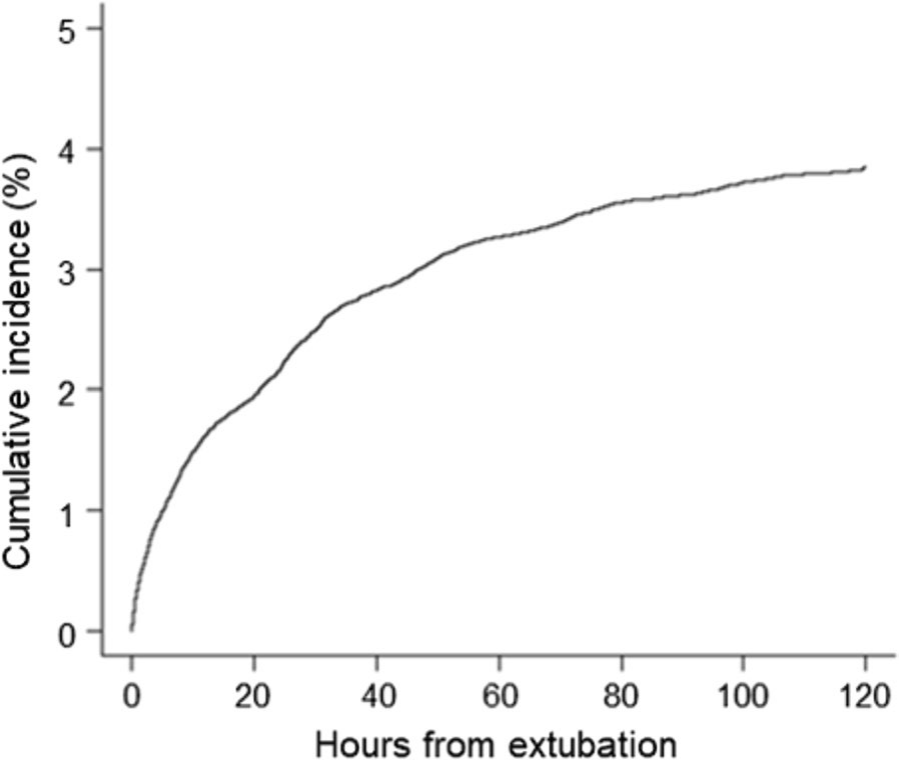
Cumulative probability of reintubation among all extubated patients

A total of 184,705 patients were admitted to the 75 ICUs during the study period. Except for 10,750 patients aged < 18 years, 8108 patients who were readmitted to the ICUs, and 105,518 patients who did not require mechanical ventilation during their ICU stay, 60,689 patients received mechanical ventilation at their first ICU admission. Except for 2068 patients who received mechanical ventilation during tracheostomy after ICU admission, 58,621 patients underwent endotracheal intubation. Subsequently, we excluded 4453 deaths during mechanical ventilation, 4491 patients who were discharged from the ICU with mechanical ventilation, 1539 patients who underwent tracheostomy during the first mechanical ventilation episode, and 56 patients with missing data on the time of termination of mechanical ventilation. Hence, 48,082 patients in 72 ICUs were identified as extubated (Fig. 1). Among all extubated patients, 46,233 were successfully extubated without reinitiating mechanical ventilation within 120 h after extubation, resulting in 1849 (3.8%) patients who required reintubation. The cumulative incidence of reintubation was described in Fig. 2**,** considering the competing risk of death using Gray’s method.

Among the total number of extubated patients, 30,703 (63.9%) were male, their median age was 70 years (IQR 60–77 years) (Additional file 1: Table S1), and the median APACHE III score was 62 (IQR 50–78); a median of 17.8 h (IQR 10.1–60.1 h) of mechanical ventilation was performed. The ICU mortality rate was 1.2%, and the in-hospital mortality rate was 5.5% (Additional file 1: Table S2). Univariable Cox proportional hazards analysis showed that reintubated patients had a higher risk of ICU and in-hospital mortality than those who did not require reintubation (*p *< 0.001 for both) (Additional file 1: Table S3). After adjusting for potential confounders, multivariable analysis also indicated a significant association between reintubation and increased ICU and in-hospital mortality (adjusted HR 1.325, 95% CI 1.076–1.633, *p *= 0.008 and adjusted HR 1.520, 95% CI 1.359–1.700, *p *< 0.001, respectively).

### Reintubation and its timing after extubation

Patients who required reintubation were stratified into five groups according to the timing of reintubation after extubation: 1037 patients (56.1%) were reintubated within 24 h after extubation, 418 (22.6%) at 24–48 h, 198 (10.7%) at 48–72 h, 111 (6.0%) at 72–96 h, and 85 (4.6%) at 96–120 h (Table 1). Emergency admission was most common among patients who were reintubated at 96–120 h and surgical admission among those reintubated at 24–48 h. Cardiac and gastrointestinal diseases were most common in patients reintubated at 48–72 h, whereas respiratory and neurological diseases were most common in patients reintubated within 24 h. A GCS score < 8 was most common in patients who were reintubated within 24 h. The ICU treatment and duration of care are described in Table 2. Patients who were reintubated at 72–96 h most frequently received RRT and noninvasive respiratory support. Patients who were reintubated at 96–120 h had the highest frequency of VV-ECMO and the longest duration of initial and total mechanical ventilation, ICU and hospital length of stay.

**Table 1:** Patient characteristics according to classification by the timing of reintubation

	All (n = 1849)	Reintubation ≤ 24 h (n = 1037)	Reintubation 24–48 h (n = 418)	Reintubation 48–72 h (n = 198)	Reintubation 72–96 h (n = 111)	Reintubation 96–120 h (n = 85)	*P* value
Age, years	71 (60–78)	71 (58–78)	71 (61–79)	73 (65–78)	72 (65–78)	70 (63–77)	0.156
Sex, male	1176 (63.6%)	639 (61.6%)	277 (66.3%)	131 (66.2%)	78 (70.3%)	51 (60.0%)	0.184
Body mass index, kg/m^2^	22.2 (19.5–25.1)	22.3 (19.5–25.1)	21.9 (19.2–24.7)	21.9 (19.2–25.0)	22.3 (19.9–25.2)	22.2 (19.8–25.6)	0.391
Comorbidity							
Chronic heart failure	51 (2.8%)	31 (3.0%)	9 (2.2%)	6 (3.0%)	4 (3.6%)	1 (1.2%)	0.776
Chronic respiratory failure	51 (2.8%)	26 (2.5%)	13 (3.1%)	6 (3.0%)	3 (2.7%)	3 (3.5%)	0.886
Chronic liver disease	24 (1.3%)	12 (1.2%)	6 (1.4%)	1 (0.5%)	3 (2.7%)	2 (2.4%)	0.333
Malignancy	74 (4.0%)	42 (4.1%)	13 (3.1%)	7 (3.5%)	4 (3.6%)	8 (9.4%)	0.166
Immunodeficiency	160 (8.7%)	74 (7.1%)	46 (11.0%)	20 (10.1%)	11 (9.9%)	9 (10.6%)	0.131
Maintenance dialysis	129 (7.0%)	60 (5.8%)	36 (8.6%)	19 (9.6%)	9 (8.1%)	5 (5.9%)	0.168
Emergency admission	1248 (67.5%)	726 (70.0%)	267 (63.9%)	120 (60.6%)	72 (64.9%)	63 (74.1%)	0.019
Surgical type of admission	1157 (62.6%)	622 (60.0%)	285 (68.2%)	132 (66.7%)	66 (59.5%)	52 (61.2%)	0.032
Systematic diagnosis for ICU admission							
Cardiac	830 (44.9%)	465 (44.8%)	184 (44.0%)	93 (47.0%)	51 (45.9%)	37 (43.5%)	0.015
Respiratory	322 (17.4%)	191 (18.4%)	69 (16.5%)	30 (15.2%)	18 (16.2%)	14 (16.5%)	
Gastrointestinal	276 (14.9%)	119 (11.5%)	80 (19.1%)	44 (22.2%)	18 (16.2%)	15 (17.6%)	
Neurological	224 (12.1%)	150 (14.5%)	44 (10.5%)	16 (8.1%)	9 (8.1%)	5 (5.9%)	
Sepsis	36 (1.9%)	22 (2.1%)	7 (1.7%)	3 (1.5%)	1 (0.9%)	3 (3.5%)	
Trauma	65 (3.5%)	38 (3.7%)	13 (3.1%)	6 (3.0%)	4 (3.6%)	4 (4.7%)	
Metabolic	37 (2.0%)	25 (2.4%)	7 (1.7%)	0 (0%)	4 (3.6%)	1 (1.2%)	
Hematological	14 (0.8%)	8 (0.8%)	1 (0.2%)	1 (0.5%)	2 (1.8%)	2 (2.4%)	
Renal/Genitourinary	8 (0.4%)	4 (0.4%)	2 (0.5%)	1 (0.5%)	1 (0.9%)	0 (0%)	
Gynecological	20 (1.1%)	8 (0.8%)	7 (1.7%)	2 (1.0%)	2 (1.8%)	1 (1.2%)	
Other	17 (0.9%)	7 (0.7%)	4 (1.0%)	2 (1.0%)	1 (0.9%)	3 (3.5%)	
APACHE II score	20 (16–25)	20 (16–25)	20 (16–25)	20 (16–24)	20 (16–24)	20 (16–26)	0.889
APACHE III score	75 (61–94)	74 (60–95)	74 (60–94)	75 (62–93)	75 (64–94)	77 (65–96)	0.551
Data within 24 h after ICU admission							
Incidence of AKI	100 (5.4%)	49 (4.7%)	25 (6.0%)	10 (5.1%)	9 (8.1%)	7 (8.2%)	0.328
PaO_2_:FiO_2_	282.5 (187.8–392.4)	280.8 (183.1–388.2)	287.8 (198.8–393.4)	300.0 (196.3–410.6)	259.1 (176.8–396.1)	264.1 (185.4–353.9)	0.436
GCS score < 8	292 (15.8%)	193 (18.6%)	51 (12.2%)	19 (9.6%)	15 (13.5%)	14 (16.5%)	0.002

Data are presented as the median and interquartile range or as numbers (percentages)

*P* values are analyzed using the chi-square (Fisher’s exact) test or the Kruskal–Wallis test

*ICU* Intensive care unit, *APACHE* Acute Physiology and Chronic Health Evaluation, *AKI* Acute kidney injury, *GCS* Glasgow Coma Scale

**Table 2:** ICU treatment and duration of care stratified by the timing of reintubation

	All	Reintubation ≤ 24 h	Reintubation 24–48 h	Reintubation 48–72 h	Reintubation 72–96 h	Reintubation 96–120 h	*P* value
VV–ECMO	35 (1.9%)	11 (1.1%)	11 (2.6%)	5 (2.5%)	3 (2.7%)	5 (5.9%)	0.008
VA–ECMO	87 (4.7%)	47 (4.5%)	19 (4.5%)	5 (2.5%)	8 (7.2%)	8 (9.4%)	0.096
Renal replacement therapy	439 (23.7%)	209 (20.2%)	106 (25.4%)	59 (29.8%)	40 (36.0%)	25 (29.4%)	< 0.001
Noninvasive respiratory support	768 (41.5%)	384 (37.0%)	196 (46.9%)	86 (43.4%)	56 (50.5%)	46 (54.1%)	< 0.001
Duration of first mechanical ventilation, h	56.1 (17.5–130.3)	54.8 (17.2–127.6)	44.0 (17.1–116.2)	55.7 (17.4–118.1)	66.6 (22.5–145.8)	94.2 (37.3–185.2)	< 0.001
Hours from extubation to reintubation, h	19.3 (4.7–43.0)	5.8 (1.8–11.9)	31.5 (27.2–39.1)	56.8 (51.4–66.4)	80.1 (75.9–88.9)	104.8 (99.7–114.4)	< 0.001
Total duration of mechanical ventilation, h	191.4 (99.8–334.1)	178.3 (97.4–314.9)	191.0 (88.8–349.4)	226.4 (103.6–386.6)	232.4 (126.8–372.7)	259.7 (125.9–463.2)	< 0.001
Number of reintubation episodes	1 (1–1)	1 (1–1)	1 (1–1)	1 (1–1)	1 (1–1)	1 (1–1)	0.137
Tracheostomy during ICU stay	614 (33.2%)	318 (30.7%)	154 (36.8%)	68 (34.3%)	45 (40.5%)	29 (34.1%)	0.078
Length of ICU stay, days	8 (5–14)	7 (4–12)	9 (6–15)	11 (7–17.75)	12 (8–18.5)	14 (10–19)	< 0.001
Length of hospital stay, days	40 (23–68)	36 (21–61)	45 (25–73)	44 (27–68)	43 (25–80)	60 (36–96)	< 0.001

Data are presented as the median and interquartile range or as numbers (percentages)

*P* values are analyzed using the chi-square (Fisher’s exact) test or the Kruskal–Wallis test

*ICU* Intensive care unit, *VV* Venovenous, *VA* Venoarterial, *ECMO* Extracorporeal membrane oxygenation

### Timing of reintubation and patient outcomes

Regarding the primary and secondary outcomes, in-hospital and ICU mortality stratified by the timing of reintubation were investigated using a Cox proportional hazards model (Table 3). The univariable analysis showed that in-hospital mortality based on reintubation within 24 h was highest in patients reintubated at 72–96 h (crude HR 1.524, 95% CI 1.061–2.189). After adjusting for the confounding factors, patients reintubated at 72–96 h likewise presented the highest mortality rates (adjusted HR 1.528, 95% CI 1.062–2.197). As for ICU mortality, univariable and multivariable Cox proportional hazards analyses similarly demonstrated the highest mortality rate in patients reintubated at 72–96 h (crude HR 1.293, 95% CI 0.741–2.256; adjusted HR 1.334, 95% CI 0.756–2.352).

**Table 3:** Patient outcomes stratified by the timing of reintubation: cox proportional hazards model

	Reintubation ≤ 24 h	Reintubation 24–48 h	Reintubation 48–72 h	Reintubation 72–96 h	Reintubation 96–120 h
ICU mortality					
N (person-day)	74 (10,562)	38 (5540)	18 (2848)	15 (1692)	12 (1804)
Crude HR (95% CI)	1 (reference)	0.964 (0.651–1.428)	0.906 (0.540–1.519)	1.293 (0.741–2.256)	0.861 (0.462–1.604)
Adjusted HR (95% CI) *	1 (reference)	0.915 (0.610–1.371)	0.991 (0.587–1.672)	1.334 (0.756–2.352)	0.833 (0.444–1.561)
In-hospital mortality					
N (person-day)	183 (50,024)	96 (22,692)	53 (10,674)	35 (6467)	27 (5968)
Crude HR (95% CI)	1 (reference)	1.187 (0.9268–1.520)	1.385 (1.020–1.880)	1.524 (1.061–2.189)	1.303 (0.869–1.954)
Adjusted HR (95% CI) *	1 (reference)	1.154 (0.898–1.485)	1.283 (0.940–1.751)	1.528 (1.062–2.197)	1.179 (0.783–1.777)

*HR adjusted for age, sex, comorbidity of chronic heart failure, comorbidity of chronic respiratory failure, APACHE III score, PaO_2_:FiO_2_, Glasgow Coma Scale, duration of first mechanical ventilation, and use of noninvasive respiratory support in mortality

*ICU* Intensive care unit, *HR* Hazard ratio, *CI* Confidence interval, *APACHE* Acute Physiology and Chronic Health Evaluation

## Discussion

### Key findings

Reintubation was significantly associated with in-hospital and ICU mortality in critically ill patients who required mechanical ventilation and were extubated. In this multicenter cohort study, approximately half of the patients were reintubated within 24 h after extubation. In terms of the time course from extubation, the highest risk of ICU and in-hospital mortality was detected during reintubation at 72–96 h.

### Relationship with prior studies

Extubation failure resulting in reintubation is a common but critical issue in the management of patients requiring mechanical ventilation in the ICU [24]. The reasons for reintubation are multifactorial, including respiratory failure, congestive heart failure, and airway obstruction with excess secretion [25–28]. Reintubation may be attributed to worsening clinical conditions such as sepsis or persistent respiratory failure [29], whereas reintubation itself may further worsen the overall prognosis of patients. Although the incidences of cardiac arrest and death are low, hypoxia, hypotension, arrhythmias, and aspiration may occur as procedural complications of reintubation [2]. Elmer et al. reviewed prospectively collected data on intubation complications in 151 reintubated patients at a tertiary care hospital in the United States [30]. Peri-intubation complications, which were mainly hypoxia and hypotension, occurred significantly more often at reintubation than at initial intubation (13% vs. 5%). Consequently, the other single-center retrospective observational study in the United States reported that a difficult airway at reintubation was associated with increased in-hospital mortality (adjusted odds ratio [OR] 2.23, 95% CI 1.01–4.93) [2]. Moreover, reintubation has been demonstrated to result in prolonged mechanical ventilation and is associated with increased mortality and adverse events, including ventilator-associated pneumonia [8, 28, 31]. Accordingly, reintubation in critically ill patients has a high mortality rate of 17–53% [19, 30, 32]. Indeed, a significantly high mortality rate of 21.3% was observed in patients who were reintubated in this multicenter cohort, involving a variety of severities and etiologies of reintubation.

Significant deterioration may occur after extubation until the patient has been reintubated [33, 34]; therefore, appropriate reinstitution of mechanical ventilation is considered to impact patient outcomes. Regarding the time course of reintubation, the majority of reintubated patients are reintubated within 24 h after extubation [25, 35]. In the present study, as many as 56.1% of reintubated patients were reintubated within 24 h of extubation. An analysis of a large cohort in the United States reported a median time to reintubation of 15 (IQR 2–45) h after extubation [23]. Miltiades et al. reported that 91.8% of 9907 reintubations occurred within 96 h of extubation. Across subanalyses of various types of patients, ICUs, and years, the authors determined that the 96-h cutoff after extubation is an essential criterion that consistently captures approximately 90% of reintubation events. Meanwhile, the effect of the time between extubation and reintubation on the course of treatment has been examined in a few studies. Epstein et al. previously studied 74 patients in the United States who underwent reintubation within 72 h of extubation after mechanical ventilation for at least 6 h [33]. The median time from extubation to reintubation was 21 (IQR 9–46) h, and mortality increased as the time from extubation to reintubation increased. The mortality rate was significantly higher in patients who were reintubated later than 12 or 24 h after extubation compared with those who were reintubated earlier (24% vs. 51% and 30.2% vs. 58.1%, respectively). In a multivariable logistic regression analysis, the authors showed that reintubation 12 h after extubation was independently associated with increased in-hospital mortality (adjusted OR 6.0, 95% CI 2.9–12.3). Furthermore, the risk of procedural complications of reintubation itself has been documented to be significantly higher with delayed reintubation at ≥ 72 h after extubation compared with reintubation at < 72 h (OR 1.05, 95% CI 1.01–1.10) [30]. However, an Australian single-center observational study of 52 reintubated patients was unable to show an association between the time from extubation to reintubation and in-hospital mortality using a logistic regression analysis utilizing continuous variables (adjusted OR 0.99, 95% CI 0.97–1.01) [24]. The detailed risk of mortality on each day between extubation and reintubation demonstrated in this study in a large cohort raises the challenge of revising the time definition of reintubation.

### Implications of study findings

Our findings clarified the practice of reintubation in critically ill adult patients using the JIPAD nationwide database. In relation to patient outcomes, reintubation at 72–96 h after extubation was associated with the highest risk of mortality. A time definition of reintubation based on the findings of the present study will contribute to the uniformity of evidence in research and international guidelines, as well as to the observation period of patients extubated in clinical practice.

### Strengths and limitations

This study has several strengths. This multicenter cohort study included a diverse population, increasing the generalizability of the findings. Quality control measures for data management, quality assurance, and auditing were established using the database. A detailed analysis based on a large cohort with adjustment for potential confounders provided an accurate description of the association between reintubation and patient outcomes.

However, our study has some limitations. First, mechanical ventilation procedures, including the decision to extubate or reintubate, were determined at the discretion of the clinicians. Weaning strategies recommended by a nationwide consensus were established in March 2015 and are widely accepted in Japan [36]. The recommended weaning protocol is as follows. Patients are considered eligible for SBT when sufficient oxygenation (SpO_2_ > 90% at FiO_2_ ≤ 0.5 and positive end-expiratory pressure [PEEP] ≤ 8 cm H_2_O) and inspiratory effort (tidal volume > 5 mL/kg; minute volume < 15 L/min; rapid shallow breathing index < 105 breaths/min/L; and pH > 7.25) are verified. Once-daily SBT with low-level pressure support (PEEP ≤ 5 cm H_2_O, pressure support ≤ 5 cm H_2_O) or T-piece at a setting of FiO_2_ < 50% for 30–120 min should be performed. SBT should be considered as failed when the patient has significant deterioration of oxygenation compared with before SBT, a respiratory rate ≥ 30 breaths/min, heart rate ≥ 140 beats/min, arrhythmia, myocardial ischemia symptoms, sustained increased blood pressure, or the appearance of respiratory distress as defined by paradoxical breathing, the use of accessory muscles, sweating, or agitation. If the patient tolerates SBT, extubation proceeds following the assessment of the risks of post-extubation upper airway obstruction and respiratory failure. If risk factors for post-extubation upper airway obstruction exist, such as prolonged intubation, a large endotracheal tube, or difficult intubation, clinicians should perform additional assessments including the cuff leak test and administer systemic steroids. Hemodynamic and respiratory parameters, including arterial blood gas, should be continuously monitored after extubation, and prophylactic non-invasive respiratory support should be considered for post-extubation respiratory failure, including respiratory fatigue. Second, our data did not provide detailed information on reintubation, including procedural complications. Reintubation may be due to multiple causes, such as post-extubation respiratory failure or impaired consciousness; however, the data do not include the reasons for reintubation. Confounding factors related to patient outcomes of reintubation, which have not been thoroughly examined, may be present. Therefore, the results of this cohort study do not provide a basis for recommending specific time periods when reintubation should be performed for improved patient outcomes. However, it does suggest that the previously considered time definition of reintubation within 48 or 72 h after extubation may be insufficient as a period of observation in terms of mortality. Recently, the use of noninvasive respiratory support has become widely available, allowing for continued respiratory management after extubation. Therefore, the time definition of extubation used in research has been extended. Conversely, the incidence of reintubation decreases over time, and the occurrence of new intubations independent of post-extubation respiratory failure cannot be exempted [37]. Therefore, a more accurate time definition of reintubation, based on the association with patient prognosis, is desirable. The results of this large cohort study provide a rationale for revising the time definition of reintubation in terms of patient outcomes and exploring more robust strategies for liberation from mechanical ventilation.

## Conclusions

In this multicenter cohort study of critically ill patients, reintubation was significantly associated with increased in-hospital and ICU mortality. The highest mortality rate was attributed to reintubation at 72–96 h after extubation. Further studies based on these findings are warranted to strengthen the evidence regarding mechanical ventilation.

### Supplementary Information

**Additional file 1: Table S1.** Patient characteristics among all extubated patients. **Table S2.** ICU treatment and patient outcomes among all extubated patients. **Table S3.** Association between reintubation and mortality: Cox proportional hazard model.

## Data Availability

The authors are contractually bound by the JIPAD project to avoid publishing or sharing data used in this manuscript.

## Abbreviations

ANZICS: Australian and New Zealand Intensive Care Society
APACHE: Acute Physiology and Chronic Health Evaluation
CI: Confidence interval
ECMO: Extracorporeal membrane oxygenation
FiO_2_: Fractional inspired oxygen
GCS: Glasgow Coma Scale
HR: Hazard ratio
ICU: Intensive care unit
IQR: Interquartile range
JIPAD: Japanese Intensive Care Patient Database
OR: Odds ratio
PaO_2_: Arterial oxygen partial pressure
PEEP: Positive end-expiratory pressure
RRT: Renal replacement therapy
SBT: Spontaneous breathing trial
VV: Venovenous
