# Supplementary material for: Time definition of reintubation most relevant to patient outcomes in critically ill patients: a multicenter cohort study
Source: Crit Care. 2023 Sep 30;27:378. doi: 10.1186/s13054-023-04668-3 (PMC10544149; doi:10.1186/s13054-023-04668-3)
Supplement: Supplementary file 1 — Additional file 1: Table S1. Patient characteristics among all extubated patients. Table S2. ICU treatment and patient outcomes among all extubated patients. Table S3. Association between reintubation and mortality: Cox proportional hazard model. [file 13054_2023_4668_MOESM1_ESM.docx]

**Table S1.** Patient characteristics among all extubated patients

|  | All (n=48,082) |
| --- | --- |
| Age, years | 70 (60–77) |
| Sex, male | 30,703 (63.9%) |
| Body mass index, kg/m^2^ | 22.6 (20.2–25.3) |
| Comorbidity |  |
| Chronic heart failure | 1,016 (2.1%) |
| Chronic respiratory failure | 564 (1.2%) |
| Chronic liver disease | 614 (1.3%) |
| Malignancy | 1,479 (3.1%) |
| Immunodeficiency | 2,664 (5.5%) |
| Maintenance dialysis | 2,728 (5.7%) |
| Emergency admission | 21,987 (45.7%) |
| Surgical type of admission | 37,630 (78.3%) |
| Systematic diagnosis for ICU admission |  |
| Cardiac | 25,901 (53.9%) |
| Respiratory | 5,365 (11.2%) |
| Gastrointestinal | 7,347 (15.3%) |
| Neurological | 4,403 (9.2%) |
| Sepsis | 542 (1.1%) |
| Trauma | 1,360 (2.8%) |
| Metabolic | 984 (2%) |
| Hematological | 119 (0.2%) |
| Renal/Genitourinary | 416 (0.9%) |
| Gynecological | 985 (2%) |
| Other | 660 (1.4%) |
| APACHE II score | 16 (13–20) |
| APACHE III score | 62 (50–78) |
| Data within 24 hours after ICU admission |  |
| Incidence of AKI | 1,247 (2.6%) |
| PaO_2_:FiO_2_ | 325.0 (228.3–422.5) |
| GCS score <8 | 3,991 (8.3%) |
| Data are presented as the median and interquartile range or as numbers (percentages).  ICU, intensive care unit; APACHE, Acute Physiology and Chronic Health Evaluation; AKI, acute kidney injury; GCS, Glasgow Coma Scale | |

**Table S2**. ICU treatment and patient outcomes among all extubated patients

|  | All (n=48,082) |
| --- | --- |
| VV–ECMO | 170 (0.4%) |
| VA–ECMO | 560 (1.2%) |
| Renal replacement therapy | 3,993 (8.3%) |
| Noninvasive respiratory support | 907 (1.9%) |
| Duration of first mechanical ventilation, hours | 17.5 (9.8–53.0) |
| Total duration of mechanical ventilation, hours | 17.8 (10.1–60.1) |
| Tracheostomy during ICU stay | 785 (1.6%) |
| Length of ICU stay, days | 2 (1–3) |
| ICU mortality | 589 (1.2%) |
| Length of hospital stay, days | 20 (13–33) |
| In-hospital mortality | 2,653 (5.5%) |
| Data are presented as the median and interquartile range or as numbers (percentages).  ICU, intensive care unit; VV, venovenous; VA, venoarterial; ECMO, extracorporeal membrane oxygenation | |

**Table S3.** Association between reintubation and mortality: Cox proportional hazard model

|  | Number of outcomes  N (person-day) | Crude HR  (95% CI) | P value | Adjusted HR  (95% CI) * | P value |
| --- | --- | --- | --- | --- | --- |
| ICU mortality |  |  |  |  |  |
| Non-reintubation | 432/116,696 | Reference |  | Reference |  |
| Reintubation | 157/22,446 | 1.580  (1.268–1.968) | <0.001 | 1.325  (1.076–1.633) | 0.008 |
| In-hospital mortality |  |  |  |  |  |
| Non-reintubation | 2,259/1,277,818 | Reference |  | Reference |  |
| Reintubation | 394/95,825 | 2.134  (1.913–2.381) | <0.001 | 1.520  (1.359–1.700) | <0.001 |
| * HR adjusted for age, sex, comorbidity of chronic heart failure, comorbidity of chronic respiratory failure, APACHE III score, PaO_2_:FiO_2_, Glasgow Coma Scale, duration of first mechanical ventilation, and use of noninvasive respiratory support in mortality.  HR, hazard ratio; CI, confidence interval; ICU, intensive care unit; APACHE, Acute Physiology and Chronic Health Evaluation | | | | | |
